# Ghost Cells as a Two‐Phase Blood Analog Fluid —Fluorescent Mechanical Hemolysis Detection

**DOI:** 10.1111/aor.15061

**Published:** 2025-07-24

**Authors:** Benjamin J. Schürmann, Bennet F. Holst, Pia Creutz, Thomas Schmitz‐Rode, Ulrich Steinseifer, Johanna C. Clauser

**Affiliations:** ^1^ Department of Cardiovascular Engineering Institute of Applied Medical Engineering, University Hospital RWTH Aachen University Aachen Germany; ^2^ Institute of Applied Medical Engineering, University Hospital RWTH Aachen University Aachen Germany

**Keywords:** fluorescent mechanical hemolysis detection, resealed ghost cells, translucent two‐phase blood analog fluid

## Abstract

**Background:**

This study investigated fluorescent hemolysis detection as an optical method to detect local hemolysis in mechanical circulatory support systems, addressing the limitations of standard hemolysis tests and current simulation methods. Standard tests, per ASTM1841‐19, quantify general hemolysis but do not localize it.

**Methods:**

We employ a two‐phase blood analog fluid composed of calcium‐loaded ghost cells and phosphate‐buffered saline. Ghost cells are hemoglobin‐depleted red blood cells, allowing for optical measurements. A calcium‐sensitive fluorescent indicator (Cal590 potassium salt, AAT Bioquest, Pleasanton, USA), activated by calcium released upon ghost cell hemolysis, enables fluorescent hemolysis detection. Hemolysis tests were conducted using porcine whole blood and the blood analog fluid, confirming that both undergo mechanical hemolysis in the Food and Drug Administration pump model.

**Results:**

The results revealed increased fluorescence intensity in response to hemolysis, with a quantitative fluorescence increase of 8.85/min at 3500 rpm and 2.5 L/min, indicating hemolysis, particularly at the rotor tip. Through image processing of fluorescence images, local hemolysis was visualized.

**Conclusion:**

This study is the first to use fluorescent hemolysis detection for local detection of mechanical hemolysis. Further refinement may enhance the design of mechanical circulatory support systems and bridge simulation limitations with experimental, localized hemolysis detection.

## Graphical abstract

Hemolysis is locally resolved in the Food and Drug Administration pump model within an experimental particle image velocimetry setup with the use of ghost cells as a two‐phase blood analog fluid.

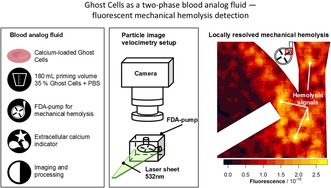

**Abbreviations**
BAF: blood analog fluid
EDTA: ethylenediaminetetraacetic acid
FDA: Food and Drug Administration
FHD: fluorescent hemolysis detection
GC: ghost cells
HCT: hematocrit
MCS: mechanical circulatory support systems
PBS: phosphate‐buffered saline
pfHb: free plasma hemoglobin

## 1 Background

Hemolysis and thrombosis are the primary challenges associated with mechanical circulatory support systems (MCSs). Hemolysis in MCSs is typically assessed via the standard hemolysis test, as outlined in ASTM1841‐19 [1]. This test involves running the MCS with human or animal blood for several hours, followed by measuring the free plasma hemoglobin (pfHb) released by red blood cells (RBCs) when damaged. However, this type of test can only determine a quantitative global increase in hemolysis but not local hemolysis.

Various simulation methods have been developed to support the assessment of local hemolysis. Most commonly used simulation methods are shear stress‐based power law models; these methods predict hemolysis by examining the relationship between shear stress and exposure time, which helps identify hemolysis‐inducing regions within the MCS [2, 3, 4, 5, 6, 7, 8, 9]. While simulation methods are based on experiments with well‐defined shear stress conditions [10] and are generally used for relative comparisons [11], quantitative validation as well as local validation in complex flow structures are missing. A method to determine local hemolysis is needed to bridge the gap between widely used simulation methods and the standard hemolysis test. We are working on fluorescent hemolysis detection (FHD), an optical method to detect local hemolysis. For FHD, we utilized a two‐phase blood analog fluid (BAF) composed of calcium‐loaded ghost cells (GCs) suspended in phosphate‐buffered saline (PBS). GCs are RBCs that have been depleted of hemoglobin, making them translucent and suitable for optical measurements such as FHD. The key of FHD is an extracellular calcium indicator, which is activated by the calcium in the GCs upon their hemolysis. FHD has been previously validated for chemically induced hemolysis by Jansen et al. [12]. The transfer from chemical to mechanical hemolysis in MCS, which is comparable to the standard hemolysis test, necessitates large volumes of BAF made from GCs. An increase in the production of GCs was recently reported by Schuermann et al. [13], enabling us to test the FHD for mechanical hemolysis in MCS.

The MCS in vitro test setup used for this study is the FDA pump (Food and Drug Administration), a generic centrifugal blood pump that has been extensively simulated and validated via particle image velocimetry (PIV) and standard hemolysis tests [14, 15, 16, 17, 18]. For the FDA pump, simulation methods suggest that the bifurcation at the diffuser and the rotor are hemolysis‐inducing regions [19].

In this study, we evaluated the hemolysis properties of our BAF in comparison to those of whole blood via hemolysis tests. Furthermore, we visualized mechanical hemolysis in the FDA pump via FHD.

## 2 Methods

Hemolysis tests were conducted with porcine whole blood and a BAF adjusted to a hematocrit (HCT) of 35% with PBS (Bio&Sell GmbH, Feucht, Germany). The BAF is mixed with GCs produced following the production method outlined by Schuermann et al. [13]. The only change in the production method is the replacement of the hemolysis solution in the third lysis with a calcium‐citrate solution (0.83 g/L CaCl_2_ and 106.1 g/L Na_3_Cit in demineralized water). This results in GCs with approximately 5.6 mmol/L inner cellular calcium. On the day of use, the GCs are washed twice in PBS and separated after centrifugation at 4000 *g* at 4°C for 60 min (Rotina 420 R, Andreas Hettich GmbH, Tuttlingen, Germany). The PBS for the BAF mixture is set to 300 mOsmol/L and 7.4 pH, and 0.1 g/L calcium‐sensitive fluorescent indicator (Cal590 potassium salt, AAT Bioquest, Pleasanton, USA) is added. Ethylenediaminetetraacetic acid (EDTA, Carl Roth GmbH, Karlsruhe, Germany) is added to bind to extracellular calcium. The binding affinity for calcium of EDTA exceeds that of the indicator, thereby adding EDTA binds calcium from the indicator and reduces the fluorescence of the indicator. The EDTA concentrations are adjusted with a stock solution of 0.2 g/L EDTA dissolved in PBS.

The required EDTA concentration varies between the experiments and is determined for each BAF mixture. The concentration needs to be close to the extracellular calcium level to prevent indicator pre‐activation and avoid excess free EDTA. We expect the optimal EDTA concentration to produce the highest signal intensity in FHD. While real‐time measurement of extracellular calcium is not possible, we determine the optimal EDTA concentration by measuring the fluorescence intensity of the mixture in a 96‐well microplate reader (Tecan Spark, Tecan Trading AG, Switzerland). Triplicates of 100 μL of GCs and 100 μL of PBS with increasing proportions of EDTA‐PBS (0–60 μL) are pipetted into the wells. The fluorescence intensity of the indicator is measured at 590 nm and an excitation wavelength of 530 nm with multiple measurements over a time span of 44.6 h after pipetting. The optimal EDTA concentration is defined as the lowest concentration that decreases more than 95% of the fluorescence intensity.

### 2.1 Hemolysis Test

Hemolysis tests are conducted for five batches of BAF and whole blood, respectively, followed by visualization tests for mechanical hemolysis. For this study, the standard hemolysis tests (ASTM1841‐19 [1]) are modified by reducing the volume to 180 mL, as described by Woelke et al. [20], and by reducing the test duration to 120 min, following the work of McNamee et al. [21]. Reducing the test volume and duration allows multiple hemolysis tests to be performed with the same pump on the same day. The FDA pump is set to 3500 rpm and a flow rate of 2.5 L/min, referred to as “flow condition #2” in previous studies [15]. Samples are taken every 20 min, from which plasma is separated from the blood at 1500 *g* for 10 min for whole blood and at 15 000 *g* for 15 min for GCs owing to their lower density. Hemolysis is measured in plasma via the cyanide method to determine pfHb [22]. Significance between pfHb_norm_ development is analyzed with a t‐test for independent regressions.

For the BAF, the samples are also tested for fluorescence intensity and extracellular calcium content. The fluorescence is measured with 200 μL of sample in a microplate reader, and the calcium concentration is measured via optical emission spectrometry with inductively coupled plasma (ICP–OES, Spectroblue, Spectro Analytical Instruments GmbH, Germany).

### 2.2 Fluorescent Hemolysis Detection

Hemolysis visualization with the BAF is performed via FHD in a PIV setup similar to that used in the studies conducted by Malinauskas et al. [15]. The experimental circuit used consists of an FDA pump, a blood bag, tubing, and a clamp. The FDA pump is arranged under a Nd:YAG laser (EverGreen2, Lumibird SA, Lannion, France) with a wavelength of 532 nm and a camera (FlowSense EO, Dantec Dynamics A/S, Skovlunde, Denmark) equipped with a 550 nm long‐pass filter. The laser is positioned to illuminate the plane of the moving rotor from the side of the diffuser with the camera orthogonal to this plane. This camera and laser position is referred to as “CS #2” in previous studies [15] and visualizes possible hemolysis‐inducing regions such as the bifurcation at the diffuser and the rotor. The camera, laser, and rotor positions are synchronized via a timing box to capture a series of 400 double‐frame images at 15 Hz and an exposure time of 30 μs. Data processing is conducted in MATLAB (MathWorks, Natick, USA), where the double‐frame images are merged to a single image for further image processing.

The circuit was primed with 80 mL of GCs and 56 mL of EDTA‐free PBS and mixed at a low speed (400 rpm, 0.1 L/min). To obtain measurements at different EDTA concentrations, different volumes of EDTA‐PBS are added sequentially, resulting in a total EDTA‐PBS volume of 29, 30.5, 34.5, and 36 mL. For each concentration, multiple image series are recorded at the operating point of 3500 rpm. In each image, the laser excited the calcium‐sensitive fluorescent indicator (Cal590 potassium salt, AAT Bioquest, Pleasanton, USA), while the 550 nm long‐pass filter attached to the camera blocked this laser light. The indicator shifted the wavelength of the laser to a non‐filtered range, allowing it to be captured by the camera. The fluorescence intensity has a logarithmic relationship with the local calcium concentration. The mean fluorescence intensity is calculated in MATLAB to track changes over time.

The single images of the image series with the highest increase in mean fluorescence are *z*‐standardized. In *z*‐standardization, the local image value is subtracted by the local mean across all images for one image series and then divided by the standard deviation of the same images. The *z*‐standardization filters out the left‐to‐right fluorescent gradient in the images and highlights areas with locally increased fluorescent signals.

A further processing of more images for a more reliable local hemolysis result involves image processing with several steps:

1. Each 2072 × 2072 pixel image is simplified to a 74 × 74 raster image by calculating local mean values from segments of 28 × 28 pixels to neglect very small bright artifacts and to reduce calculation intensity for the processing.
2. Each raster image is normalized by its image mean value to account for differences in laser pulse intensity between pulses.
3. Each pixel in raster images is *z*‐standardized by subtracting this pixel mean value over all images of the same EDTA concentration and then divided by the standard deviation.
4. The sum of the raster images is calculated per EDTA concentration.
5. All positive raster values are summed across different EDTA concentrations.
6. Finally, the resulting images are processed with a gaussian filter with a filter size of 5 and a standard deviation of 2 to highlight the areas of hemolysis for data presentation.

## 3 Results

### 3.1 Optimal EDTA Concentration

**FIGURE 1:**
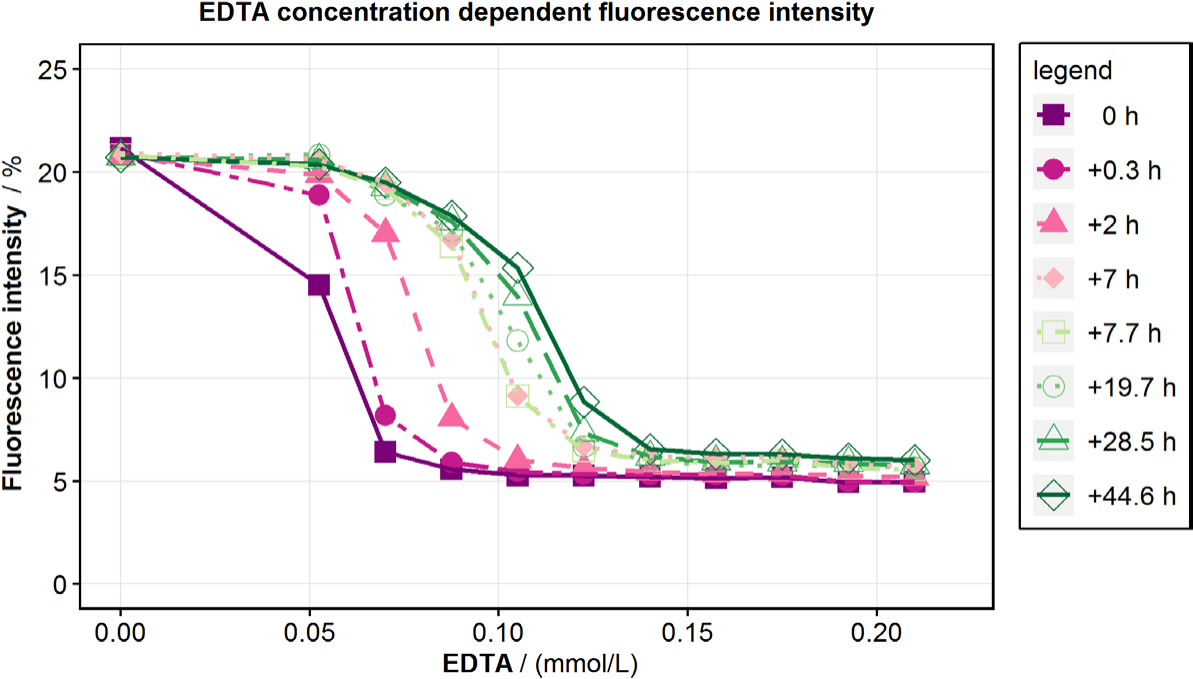
EDTA concentration‐dependent fluorescence intensity. The fluorescence intensity was measured in a 96‐well microplate reader (Tecan Spark, Tecan Trading AG, Switzerland) at different time points after mixing 100 μL of GC with 100 μL of PBS with increasing EDTA concentrations. [Color figure can be viewed at wileyonlinelibrary.com]

The fluorescence signal in determining the optimal EDTA concentration depended on both EDTA concentration and time (Figure 1). The optimal EDTA concentration is defined as the point at which the indicator's fluorescence is reduced by 95%. Within the first 7 h after pipetting, this optimal concentration increased by 0.05 mmol/L. Therefore, in the hemolysis tests, the EDTA concentration is increased by 0.05 mmol/L to ensure that the optimal concentration is maintained throughout the experiment duration. For FHD visualization, the optimal EDTA concentration is used to estimate the initial amount of EDTA‐PBS added.

### 3.2 Hemolysis Tests

**FIGURE 2:**
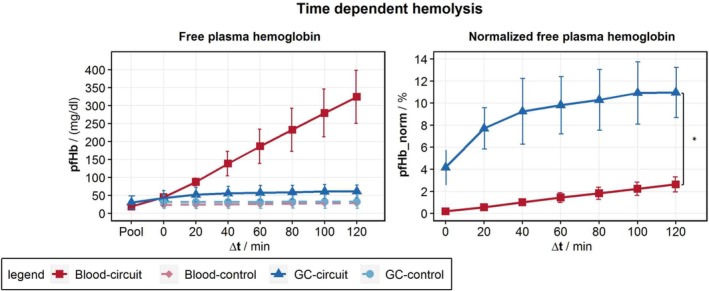
Time‐dependent hemolysis of the hemolysis tests is demonstrated as free plasma hemoglobin in the hemolysis test with a blood analog fluid made from ghost cells (GCs) and porcine whole blood. Normalized free plasma hemoglobin accounts for the different hemoglobin concentrations in blood and GCs. It increases significantly (*p* < 0.05) faster for GCs compared with blood. [Color figure can be viewed at wileyonlinelibrary.com]

Hemolysis tests revealed an increase in pfHb in the whole blood over time as estimated (Figure 2). Since GCs are hemoglobin‐depleted, the absolute increase in pfHb is distinctly lower compared with whole blood. For better comparison, we normalize different Hb levels in GCs and whole blood with the free plasma hemoglobin in the control (pfHb_control_) and total hemoglobin (Hb_total_) levels [1].

(1) $${\text{pfHb}}_{\mathrm{norm}}\left(t\right)=\left(\frac{\text{pfHb}\left(t\right)-{\text{pfHb}}_{\text{control}}\left(t\right)}{{\mathrm{Hb}}_{\text{total}}}\right)$$

This normalized pfHb indicates a pronounced increase in hemolysis for the GCs during the initial phase, and a slower increase from 40 min of experimentation on. Owing to malfunctions in the pump, we had to exclude some hemolysis tests from the data processing, leaving us with only three tests for whole blood and BAF, respectively.

**FIGURE 3:**
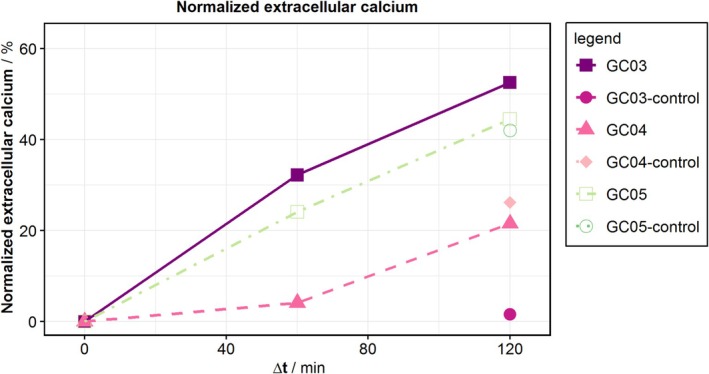
Normalized extracellular calcium of the hemolysis tests. The calcium concentration was measured via optical emission spectrometry with inductively coupled plasma (ICP–OES, Spectroblue, Spectro Analytical Instruments GmbH, Germany). [Color figure can be viewed at wileyonlinelibrary.com]

**FIGURE 4:**
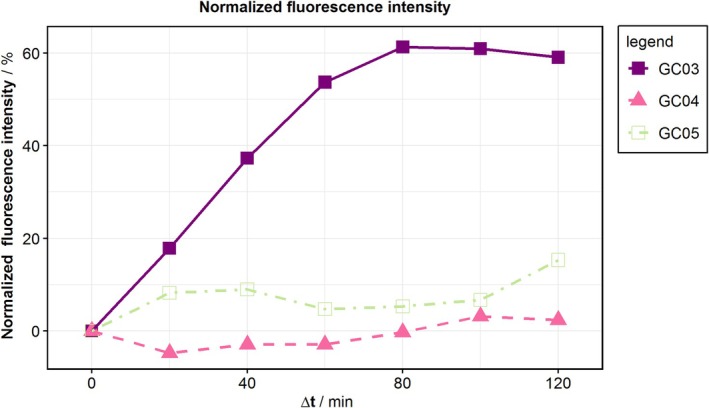
Fluorescence intensity of the hemolysis tests. The fluorescence was measured with 200 μL of sample in a 96‐well microplate reader (Tecan Spark, Tecan Trading AG, Switzerland). [Color figure can be viewed at wileyonlinelibrary.com]

While all calcium measurements reveal an increase in extracellular calcium (Figure 3), in only one experiment, GC03, the control has a lower level of extracellular calcium. This experiment is also the only experiment demonstrating an increase in fluorescence (Figure 4), with a strong increase in the first hour, after which a plateau is reached.

### 3.3 Fluorescent Hemolysis Detection

**FIGURE 5:**
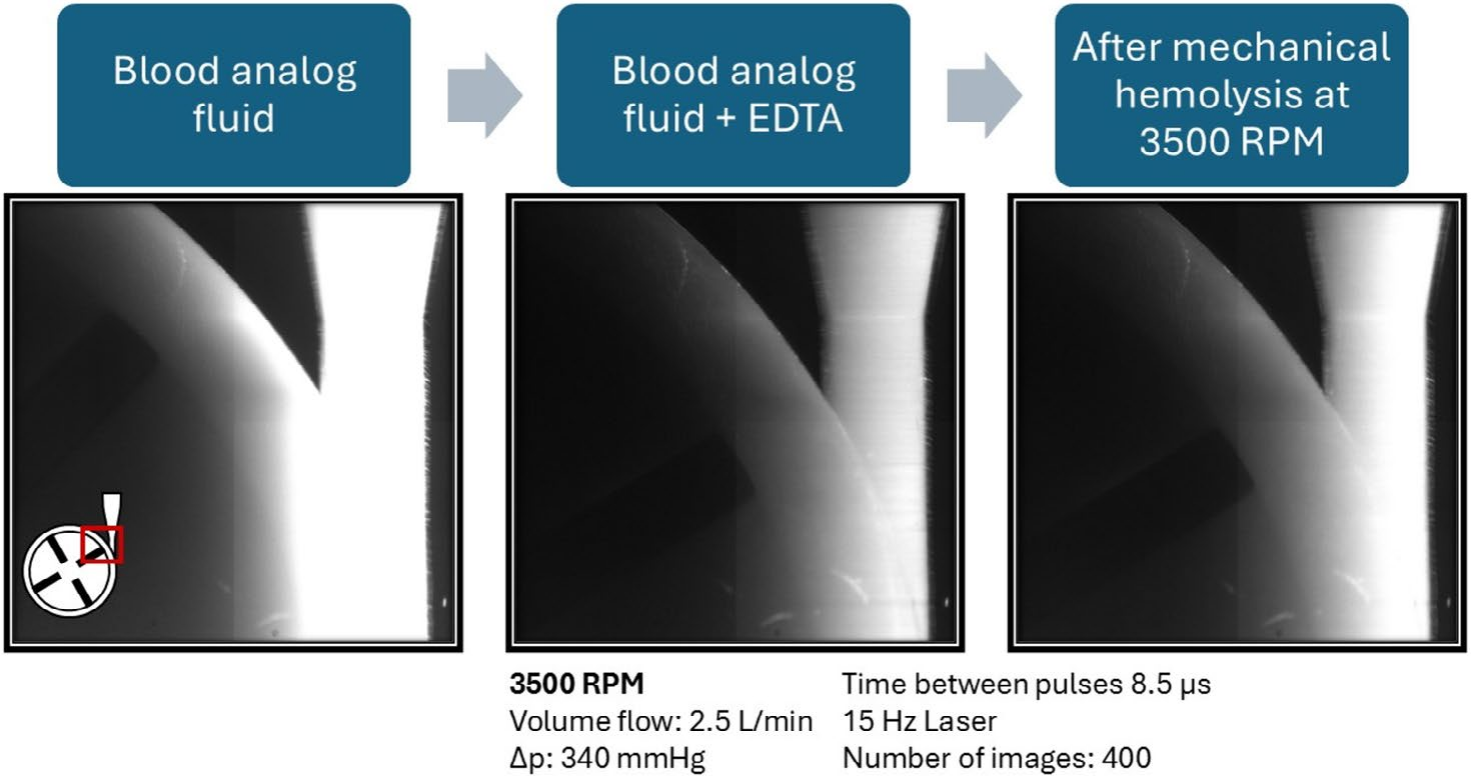
Images for fluorescent hemolysis detection before adding EDTA, before hemolysis on the FDA pump, and after mechanical hemolysis at a pump speed of 3500 rpm. [Color figure can be viewed at wileyonlinelibrary.com]

**FIGURE 6:**
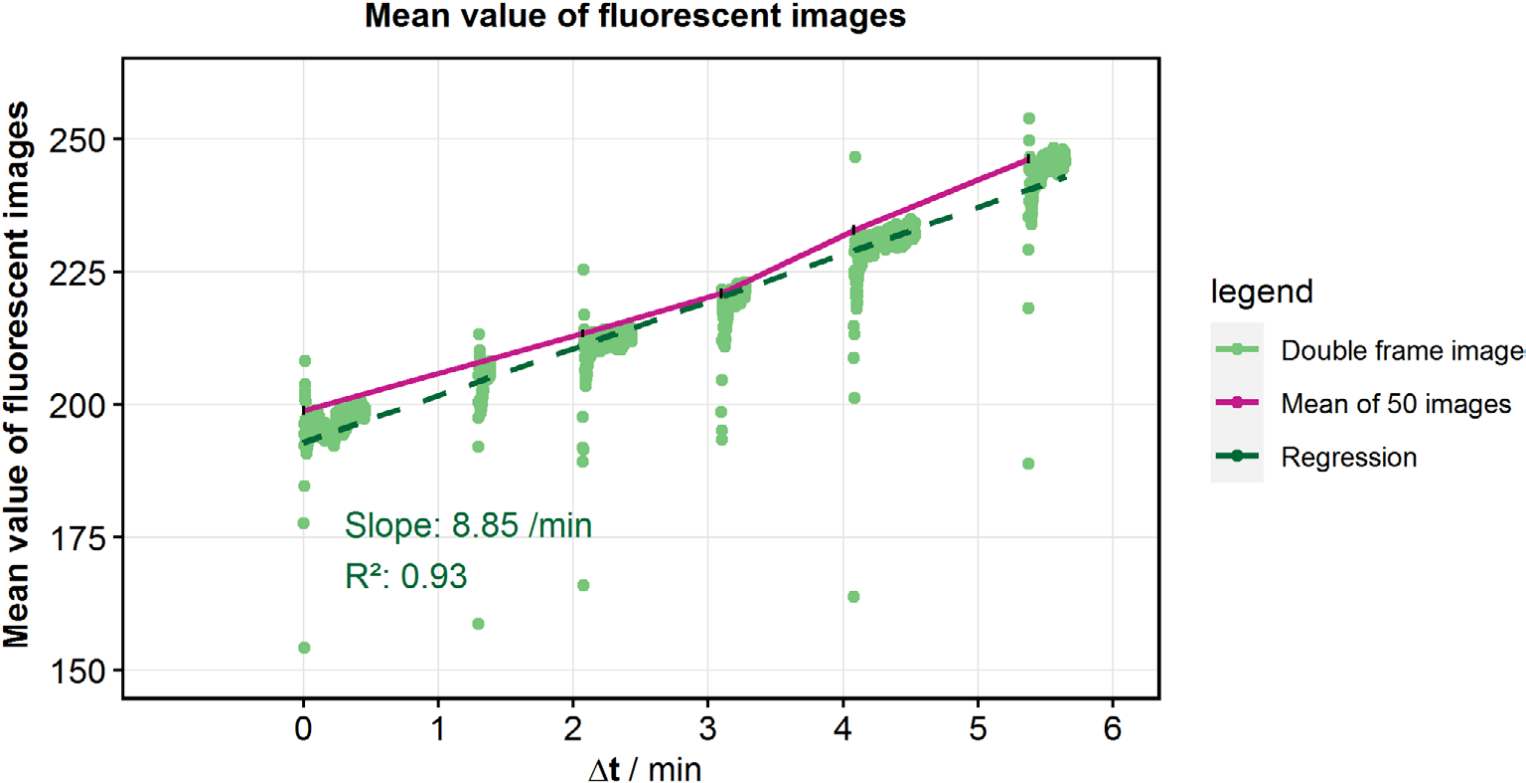
Mean value of fluorescence images taken with fluorescent hemolysis detection. The image mean values for 1198 images are calculated from the added double‐frame images. The mean of multiple images is then calculated from the last 50 images of one series. For this run at 3500 rpm, an increase in fluorescence of 8.85/min was measured. [Color figure can be viewed at wileyonlinelibrary.com]

**FIGURE 7:**
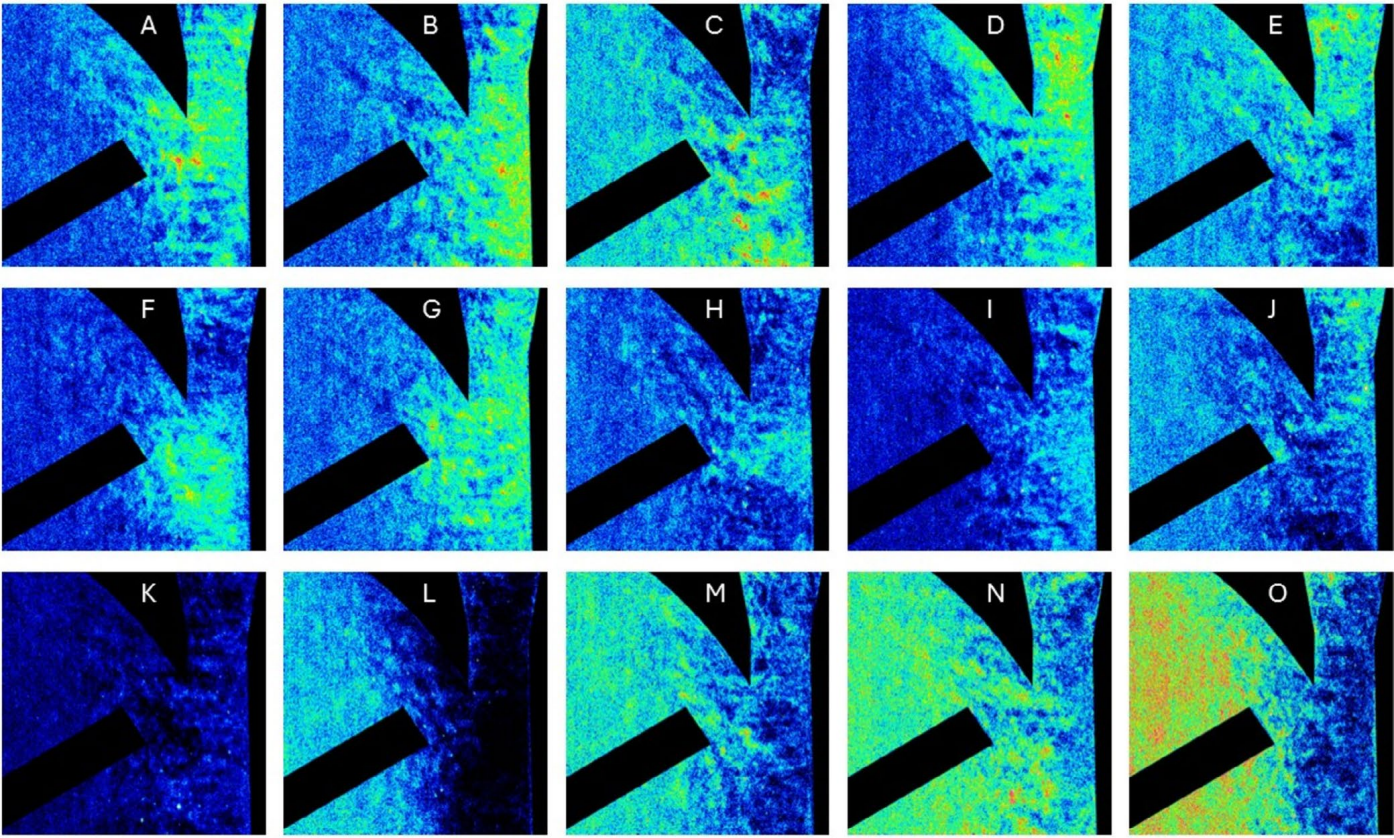
Fluorescence hemolysis detection on single images with mean‐based *z*‐standardization. Panels (A–G) show selected single images that visually demonstrate expected FHD signal patterns, with localized brightness increases at the rotor tip or within the diffuser region, consistent with hemolysis hotspots. Panels (H–J) display similar but less pronounced signal intensities. In contrast, panels (K–O) highlight the limitations of single‐image analysis: Variations in laser intensity and noise result in inconsistent regions being falsely highlighted. [Color figure can be viewed at wileyonlinelibrary.com]

**FIGURE 8:**
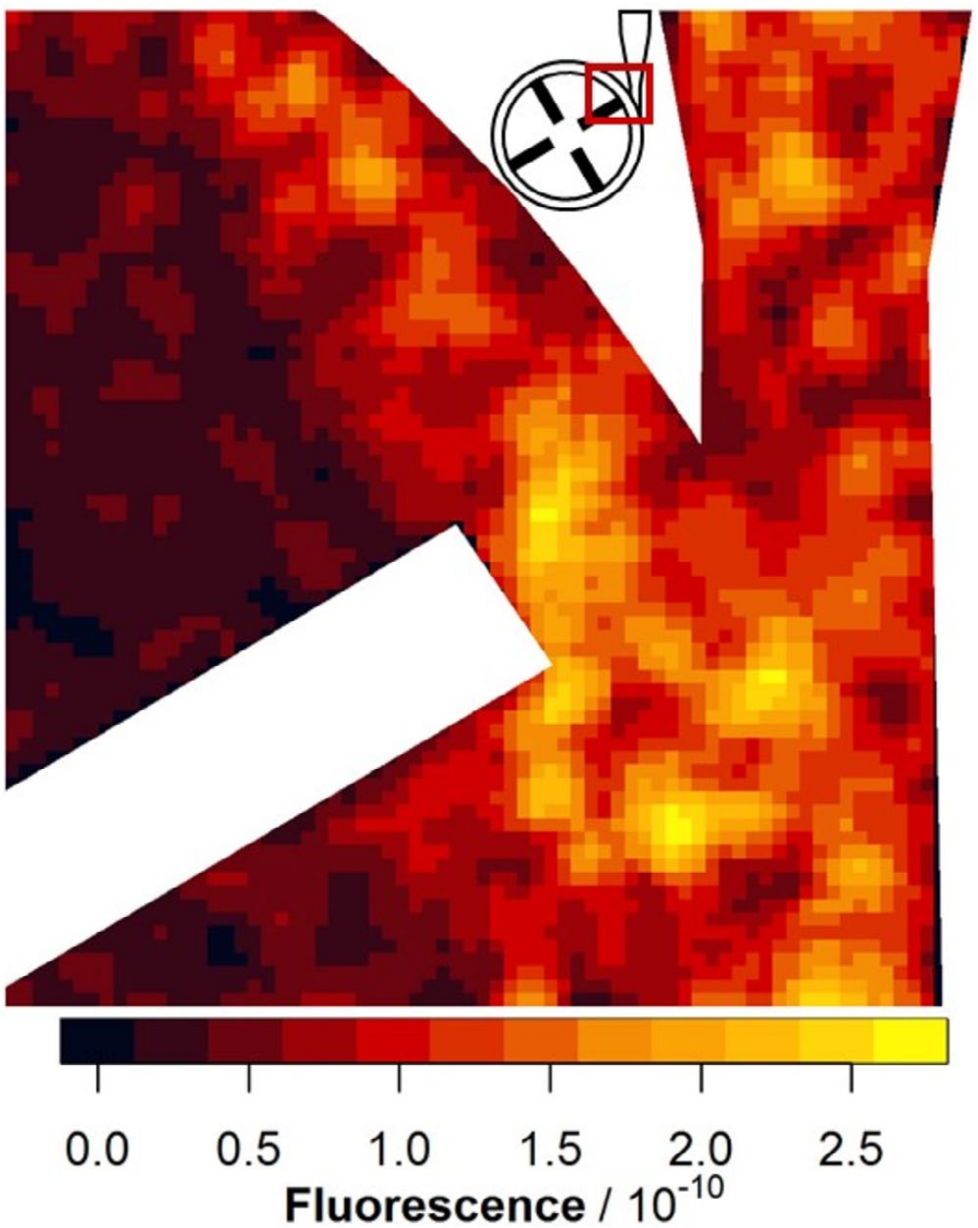
Fluorescence hemolysis detection resolved locally after image processing. A total of 6761 images were processed by reducing them to a 74 × 74 raster, dividing each image by its own mean value, *z*‐standardizing the images with the same EDTA concentration, and adding all image values per raster together. The results for each individual EDTA concentration are added in the Appendix S1. [Color figure can be viewed at wileyonlinelibrary.com]

Example images of the FHD are shown in Figure 5. In each image, the laser excitation comes from the right side, leading to a fluorescence gradient. Possible hemolysis‐inducing regions, such as the diffuser, the rotor, and some bright artifacts, are visible. Furthermore, the overall fluorescence decreases after the addition of EDTA and then increases again after mechanical hemolysis. This mechanical hemolysis at 3500 rpm and 2.5 L/min is quantitatively characterized by an increase in the mean value of the fluorescence images by 8.85/min with a coefficient of determination of 0.93. This is the largest increase in fluorescence measured with 29 mL of EDTA‐PBS for 1198 images (Figure 6). A selection of images from the image series with the highest increase in mean fluorescence is presented in Figure 7. For a more reliable local hemolysis result, 6761 images are processed, and the result is shown in Figure 8. The image processing steps neglect the brightness gradient as well as bright artifacts. The left third of the image, where the absorption is the highest, shows no signal. The highest fluorescent signal indicating the location of hemolysis is at the tip of the moving rotor and another one at the expansion of the diffuser.

## 4 Discussion

The hemolysis test confirmed that the GCs as well as the RBCs in whole blood are hemolyzed by the FDA‐Pump. The normalized pfHb increases significantly (*p* < 0.05) faster for GCs compared with RBCs (Figure 2). This applies in particular to the filling process as well as the first 40 min into the hemolysis test. We link this to the predisposition of the GCs due to their production process the day before testing. Additionally, a nonhomogeneous predisposition of the GC population as a result of the production process may contribute to the nonlinear hemolysis trend over time. Further, the high inner cellular calcium concentration may also cause the nonlinear hemolysis trend due to its reducing effect on cell deformability [23].

Compared with other BAFs, GCs are the only fluid that can be used for testing hemolysis because they have only translucent RBC substitutes [24], which can be hemolyzed in the shear stress range of the MCS. We chose to use normalized pfHb instead of the commonly used modified index of hemolysis (MIH), which incorporates both hemoglobin (Hb) and hematocrit (HCT) [1]. Previous studies by Schuermann et al. [13] show that measuring HCT in GCs is prone to errors, which would result in errors in the MIH.

The extracellular calcium in the BAF was expected to be an equivalent for pfHb in whole blood, but only in one of the experiments, GC03, a calcium increase compared with that of the control was measured (Figure 3). We found different explanations for this variation compared with pfHb. First, the calcium channels in the cell membranes are still active, and some calcium can escape without hemolysis, leading to an increase in calcium even in the nonhemolyzed control. Second, calcium binds to both intact and damaged cell membranes, and centrifugation removes these calcium‐bound membranes from the plasma. Third, the calcium measurement requires larger plasma volumes than those available from our circuit and control, leading to dilution for the measurements and decreased measurement sensitivity.

The fluorescence intensity only increased for experiment GC03 within the first hour (Figure 4). A reason for the lack of increase in other experiments could be under‐ or overestimation of the optimal EDTA concentration. To avoid this in the FHD measurements, we added EDTA sequentially. An increase in fluorescence intensity within the first hour of the experiment also aligns with the pronounced increase in normalized pfHb for GCs. Therefore, in the FHD measurements, we limit the exposure to the operation point by using a lower speed of 400 rpm to increase mixing between the measurements and attempt to reach the optimal EDTA concentration within the first hour of running at the operation point.

### 4.1 Fluorescent Hemolysis Detection

The images generated in the FHD have a fluorescence gradient from the laser facing side, degrading away from the laser (Figure 5). This is due to the remaining light absorbance and scattering of the GCs. Therefore, the darker third is dominated by noise in further image processing. The optimal EDTA concentration decreases the fluorescence of the images, and it increases again after applying mechanical hemolysis. This increase in mean image fluorescence is calculated to be 8.85/min (Figure 6). While visually compelling FHD results can be obtained from individual images using simple *z*‐standardization (Figure 7), such an approach lacks the statistical robustness required for scientific interpretation. Therefore, as described in the methods section, we implemented a multistep image processing pipeline capable of aggregating and standardizing thousands of images (Figure 8). This more rigorous approach underpins the validated FHD results of image processing showing a high FHD signal at the tip of the moving rotor and thereby presents a local detection of mechanical hemolysis for the first time.

To complement these findings, the corresponding data separated by EDTA concentration and processed using an alternative median‐based image analysis are shown in Figure S1. In addition, two further experiments—where the overall fluorescent signal is less pronounced—are included in the Supporting Information (Figure S2: 12 778 images, and Figure S3: 9776 images). Despite lower signal intensities, both experiments identify the same regions of interest, namely, the rotor tip and diffuser, thereby supporting the reproducibility of the spatial hemolysis pattern observed across different experimental conditions.

### 4.2 Limitations

PBS was chosen instead of plasma as the BAF base for two reasons: first, to reduce extracellular calcium levels that could interfere with the fluorescent indicator, and second, to allow experiments to be conducted the day after GC production. We acknowledge that this choice affected the rheological properties of the BAF, making it less representative of whole blood. At a shear rate of 1000/s, the BAF in the FHD had a viscosity of 1.64 mPa s compared with 2.82 mPa s in whole blood at 35% HCT [13].

For the hemolysis tests, we maintained the circuit temperature at 37°C ± 1°C, in accordance with regulatory standards. However, this requires both heating and cooling due to the heat generation of the pump. In the FHD, a steady temperature range is not possible because of the change in pump speed and heat generation and the circuit's optical accessibility with a laser and camera. During the FHD experiments, the temperature varied from 22°C to 38°C; this temperature difference may lead to more differences in the hemolysis between BAF and whole blood than previously determined in the hemolysis tests.

For image processing, we planned to use control images that can be subtracted from our operation point images, as was done for the proof of chemical hemolysis by Jansen et al. [12]. However, control images at a pump speed of 400 rpm were inconsistent, including variations in the image mean fluorescence, rotor position, and fluorescence behavior. Therefore, local hemolysis detection was performed with multiple image processing steps from the acquired images at the operation point.

Possible improvements for future usage of the FHD would be to increase the exposure time of the camera over the two laser pulses instead of acquiring double‐frame images. Furthermore, we image the same plane with the laser exciting from a different angle. Thereby, light scattering by the pump could be changed, and the determined position of local hemolysis could be validated.

## 5 Conclusion

For the first time, mechanical hemolysis is successfully visualized via FHD. Localized hemolysis detection for the FDA pump is successfully identified. When mixed with PBS, GCs provide a viable translucent two‐phase BAF capable of mimicking the hemolysis behavior of whole blood. In the future, FHD can be used to improve the design of MCS systems by bridging the gap between widely used simulation methods and quantitative standard hemolysis tests with experimental locally resolved hemolysis detection.

## Author Contributions

Schuermann: Concept, data collection, data analysis, statistics, interpretation and drafting of the article; Holst and Creutz: Data collection; Schmitz‐Rode and Steinseifer: Critical revision of the article; Clauser: Concept of article, critical revision of the article.

## Ethics Statement

The work with porcine blood from a slaughterhouse does not necessitate an ethics approval, but in‐house protocols for a controlled environment and disposal are followed.

## Conflicts of Interest

The authors declare no conflicts of interest.

## Supporting information


Figure S1
