# Supplementary material for: Ghost Cells as a Two‐Phase Blood Analog Fluid —Fluorescent Mechanical Hemolysis Detection
Source: Artif Organs. 2025 Jul 24;49(10):1540–7. doi: 10.1111/aor.15061 (PMC12625480; doi:10.1111/aor.15061)
Supplement: Supplementary file 1 — Figure S1 [file AOR-49-1540-s001.pdf]

Supplementary Material

Ghost cells as a two-phase blood analog fluid — fluorescent mechanical hemolysis detection

Benjamin J. Schürmann[1], Bennet F. Holst[1], Pia Creutz[1], Thomas Schmitz-Rode[2], Ulrich Steinseifer[1], **Johanna C. Clauser[1]**

[1] Department of Cardiovascular Engineering, Institute of Applied Medical Engineering, University Hospital RWTH Aachen University, Germany

[2] Institute of Applied Medical Engineering, University Hospital RWTH Aachen University, Germany

Corresponding Author: Clauser@ame.rwth-aachen.de

Figure I shows the results of fluorescent hemolysis detection (FHD) separated by EDTA concentrations for the primary experiment presented in the manuscript. The top row illustrates the FHD signal for each concentration without summing the results across EDTA concentrations. In contrast, the bottom row presents an alternative image processing approach in which the original 2072×2072-pixel images are down-sampled to a 74 x74 raster image by summing up 28 × 28 pixels via median values (step 1), followed by local z-standardization by subtracting the median (step 3).

Figure II presents FHD results from an additional experiment using ghost cells loaded with a lower estimated intracellular calcium concentration of 2.6 mmol/L. This experiment with a lower calcium concentration was performed to evaluate at which calcium concentration a higher FHD signal could be acquired. However, the higher concentration presented in the manuscript outperformed the lower concentration. While the absolute signal intensity is lower than in the main dataset, the same regions of interest—at the rotor tip and in the diffusor—are consistently highlighted. However, when using median-based standardization, the low baseline intensity in low-signal regions results in artificially high normalized values, especially in the rotor area, where no actual hemolysis is expected.

Figure III shows FHD results from a third experiment in which a potassium-sensitive fluorescent indicator (IPG-1, ION Biosciences, USA) was used instead of a calcium-sensitive one. Ghost cells were loaded with intracellular potassium and washed with a potassium-free saline solution. In this experiment, the overall FHD signal was weaker, the rotor tip and diffusor again appeared as consistent regions of interest. Similar to Figure II, applying median-based standardization in low-signal areas led to artificially elevated signals. In addition, a rotor-shaped artifact appeared in the result, likely due to an error in image masking.

The change in indicator-target combination was introduced to evaluate whether a stronger FHD signal could be achieved. Unlike calcium, potassium lacks a binding agent equivalent to EDTA, making it impossible to control the extracellular potassium concentration. However, because potassium is naturally more concentrated inside red blood cells (RBCs) than outside, the cell membrane's physiological ion transport mechanisms help maintain a separation between the intracellular potassium (the indicator target) and the extracellular indicator. In contrast, calcium typically has a lower intracellular concentration than extracellular concentration, requiring artificial manipulation—such as EDTA addition—to preserve this separation. Additionally, elevated calcium levels can impair cell deformability, potentially compromising the mechanical behavior of ghost cells. By using a potassium-sensitive fluorescent indicator instead, it may be possible to avoid such complications and more closely replicate the physiological properties of blood.

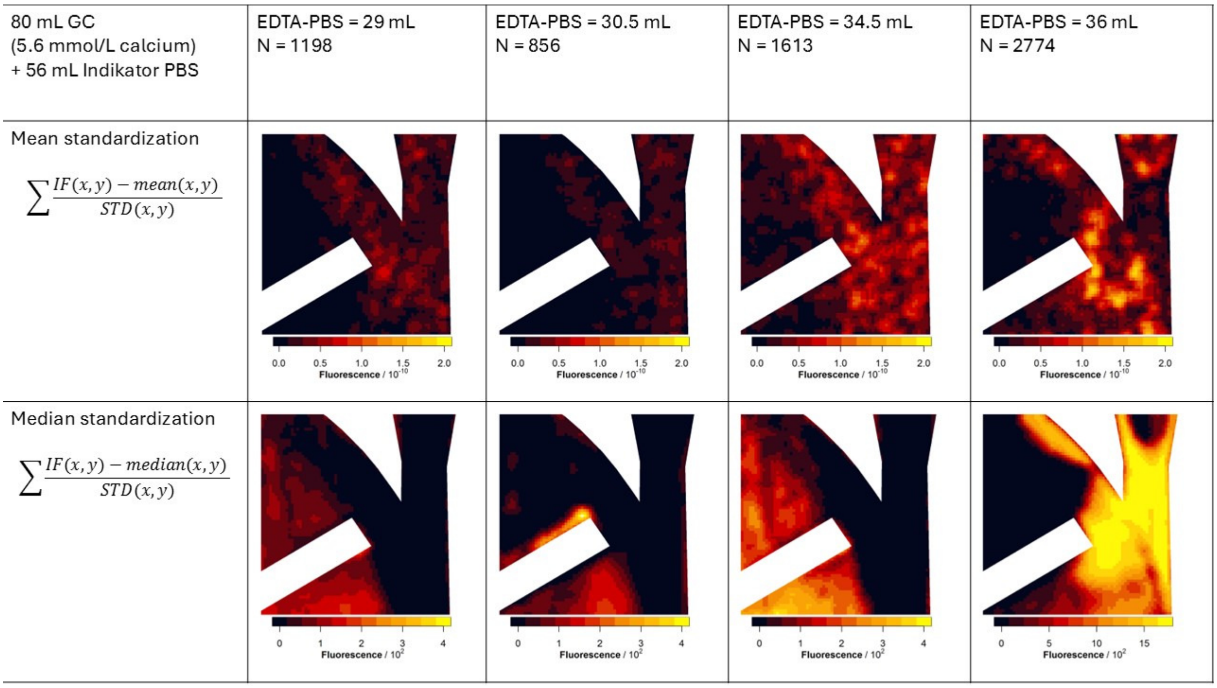

Figure I: **Fluorescence hemolysis detection separated by EDTA concentrations.** Top row: FHD signal for each EDTA concentration, calculated without applying step 5 of the image processing pipeline (summing across concentrations). Bottom row: FHD signal with altered step 1 (conversion 28×28 pixel to one pixel using local median values) and step 3 (local z-standardization by subtracting the median). Note: the color scale for image 4 (highest EDTA concentration) is adjusted due to higher signal intensity.

80 mL GC<br>(2.6 mmol/L calcium)<br>+ 52 mL Indikator PBS	EDTA-PBS = 30 mL<br>N = 3719	EDTA-PBS = 31.5 mL<br>N = 2722	EDTA-PBS = 33 mL<br>N = 2861	EDTA-PBS = 34.5 mL<br>N = 3466
Mean standardization<br>$\sum \frac{IF(x,y) - mean(x,y)}{STD(x,y)}$	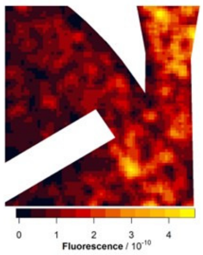	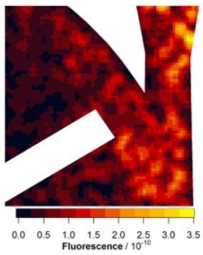	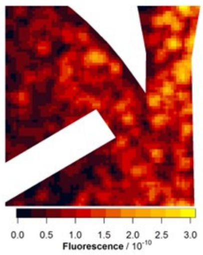	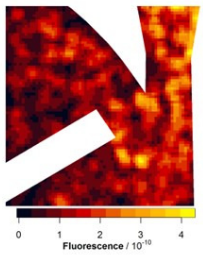
Median standardization<br>$\sum \frac{IF(x,y) - median(x,y)}{STD(x,y)}$	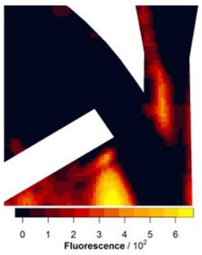	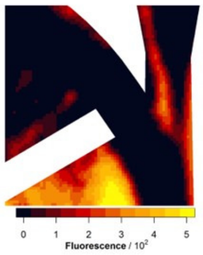	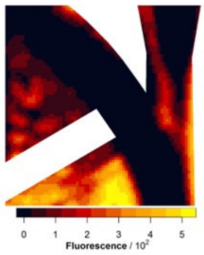	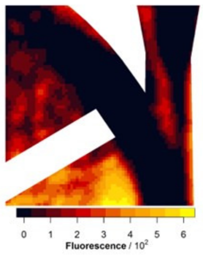

Figure II: **FHD by EDTA concentration for lower intracellular calcium.** Ghost cells were loaded with 2.6 mmol/L intracellular calcium. Using mean-based normalization, signal intensity remains visible, though less pronounced. When applying median-based standardization, the less pronounced signal lead to artificially high normalized signals in the rotor area where no real hemolysis signal is present.

64 mL GC (potassium free washing solution)<br>+ 70 mL NaCl<br>+ 150 µg IPG-1	N = 4945	N = 2559	N = 2272	
Mean standardization<br>$\sum \frac{IF(x,y) - mean(x,y)}{STD(x,y)}$	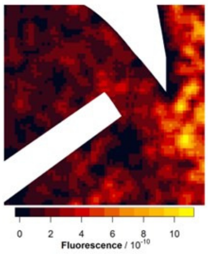	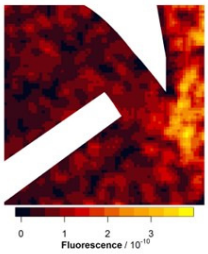	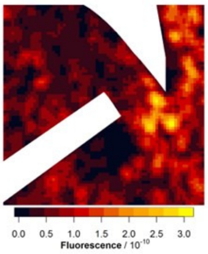	
Median standardization<br>$\sum \frac{IF(x,y) - median(x,y)}{STD(x,y)}$	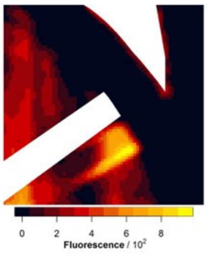	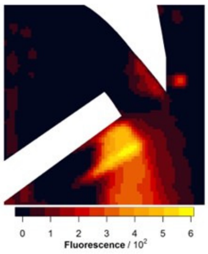	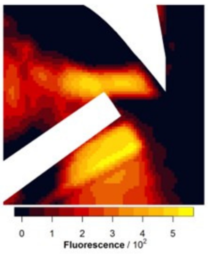	

Figure III: **FHD using a potassium-sensitive fluorescent indicator.** This experiment replaces calcium with potassium as the intracellular target. Ghost cells were prepared using a potassium-free saline solution, and the potassium-sensitive indicator IPG-1 (ION Biosciences, San Marcos, USA) was used. While FHD signal strength is lower than in the primary dataset, peak areas still align with expected regions—at the rotor tip and in the diffusor. When applying median-based standardization, the less pronounced signal lead to artificially high normalized signals in the rotor area where no real hemolysis signal is present. Also in median-based standardization, a rotor-shaped artifact appears in the result, likely caused by an error in masking during image processing.
